# Supplementary material for: Learning curve and surgical outcome of robotic assisted colorectal surgery with ERAS program
Source: Sci Rep. 2022 Nov 29;12:20566. doi: 10.1038/s41598-022-24665-w (PMC9709162; doi:10.1038/s41598-022-24665-w)
Supplement: Supplementary file 1 — Supplementary Information 1. [file 41598_2022_24665_MOESM1_ESM.zip › LSC Raw data-Ñ[▒K/108/P_ñw╝╨░O▒Kñσ.pdf]

臺中榮民總醫院 臺中市臺灣大道四段1650號 病理部活體組織[illegible] 臨床醫學實驗及病理診斷中心 檢驗(查)報告

[redacted]

Pathologic diagnosis:
1. Rectum, laparoscopic low anterior resection --- a. Status post endoscopic mucosal resection with submucosal fibrosis. No residual tumor seen. The bilateral cut ends and mesocolic/perirectal lymph nodes (0/15) are free of tumor. b. Tubulovillous adenoma and hyperplastic polyps.
2. Rectum, cut end, distal, resection --- Unremarkable.
3. IMA root, dissection --- Unremarkable fibroadipose tissue.

Prognostic and predictive factor:
(Combined with report S0938409)
1. Tumor size: 1 cm.
2. Depth of invasion: Submucosa.
3. Histologic grade: G2: Moderately differentiated.
4. Angiolymphatic invasion: Not identified.
5. Perineural invasion: Not identified.
6. Tumor deposits (discontinuous extramural extension): Not identified.
7. Macroscopic tumor perforation: Not identified.
8. Surgical Margins: a) Proximal Margin: Uninvolved by invasive carcinoma. b) Distal Margin: Uninvolved by invasive carcinoma. c) Radial or Mesenteric Margin: Uninvolved by invasive carcinoma.
9. Lymph node status: Uninvolved (0/15).
10. Extranodal involvement: Not identified.
11. Tumor regression grading S/P CCRT: No known presurgical therapy.
12. Type of polyp in which invasive carcinoma arose: Not identified.
13. Additional pathologic findings: Tubulovillous adenoma and hyperplastic polyps.
14. Pathological TNM Stage: pT1N0(According to the eighth edition, American Joint Committee on Cancer Staging Guidelines for Tumors).
15. TNM descriptors: Not applicable.

Gross description:
The specimen consists of 1) a segment of rectum, 18 cm in length and 5 cm in proximal cut end circumference, 7 cm in distal circumference, and perirectal soft tissue. Grossly, there is a post-polypectomy scar, 1x1 cm, and 3 cm away from the distal cut end, noted. No definite tumor seen grossly. There are also multiple tiny polyps, up to 0.5x0.5 cm, noted around the scar. The both cut ends are free of tumor and polyp grossly. 2) distal cut end, a piece of tan mucosa-covered soft tissue, 4x2x1 cm. 3) IMA root, a piece of tan yellow soft tissue, 2x1x1 cm. Representative part for section: A) specimen 2 B) specimen 3 C) proximal cut end D) radial margin E) polyps F-J) scar site K-M) lymph nodes.

Note:
This case has been peer reviewed by two doctors.
#T-59600_2 #M-78000_2 0 1105 SP0000
#T-C4560_2 #M-09183_2 0 1105 000000

已電子簽章

敬告：本單僅供醫師參考使用，不具任何申請效力！！
請病患妥為保管，若不慎遺失需自負個人資料外洩責任！
